# Incidence of Cholangiocarcinoma in the USA from 2001 to 2015: A US Cancer Statistics Analysis of 50 States

**DOI:** 10.7759/cureus.3962

**Published:** 2019-01-25

**Authors:** Nicolas Patel, Bikramjit Benipal

**Affiliations:** 1 Internal Medicine, New York University School of Medicine, New York, USA; 2 Internal Medicine, Temple University, Philadelphia, USA

**Keywords:** cholangiocarcinoma, gastroenterology, hepatology, epidemiology, cancer

## Abstract

Introduction

Cholangiocarcinoma is an aggressive and rare cancer of the bile duct with a very poor prognosis. It accounts for approximately three percent of gastrointestinal cancers but nearly 20 percent of deaths are from hepatobiliary cancers. Cholangiocarcinoma is also a clinically silent disease that presents at advanced stages. In this study, we wanted to identify subpopulations at the greatest risk of developing cholangiocarcinoma such that we can improve diagnosis and ultimately reduce the cancer mortality rate.

Methods

The United States Cancer Registry (USCS) was used to obtain data for cholangiocarcinoma from 2001 to 2015. Incidence analysis was done for sex, race, stage, primary location (intrahepatic bile duct or extrahepatic bile duct), and US regional location.

Results

The overall incidence of cholangiocarcinoma from 2001 to 2015 was 1.26 per 100,000 people per year. The overall incidence rates were greatest for each stratification in males, Asian and Pacific Islanders (API), distant disease, intrahepatic bile duct cholangiocarcinoma (ICC), and in the Northeast. Incidence rates were increasing between 2001 and 2015 in all subpopulations. Compared to extrahepatic bile duct cholangiocarcinoma (ECC), ICC increased significantly between 2001 and 2015. From 2001 to 2007, the annual percent change (APC) for ICC was 2.79, from 2007 to 2010 the APC was 17.02, and from 2010 to 2015 the APC was 9.67. Moreover, the incidence of distant disease also increased significantly with an APC of 9.22.

Conclusion

In our study, we analyzed the incidence of cholangiocarcinoma in all 50 states in the USA. We found that the incidence is increasing in all subpopulations and specifically at a dramatic rate for ICC and those with distant disease at the time of diagnosis. Ultimately, our findings identified at-risk populations who need closer monitoring for cholangiocarcinoma.

## Introduction

Cholangiocarcinoma is an aggressive and rare cancer of the bile duct that often presents after metastasis with a very poor prognosis [1]. It accounts for approximately three percent of gastrointestinal tumors and nearly 20 percent of deaths from hepatobiliary cancer [2]. Cholangiocarcinoma can occur anywhere in the biliary tract; however, it is subclassified anatomically as either intrahepatic bile duct cholangiocarcinoma (ICC) or extrahepatic bile duct cholangiocarcinoma (ECC) [1]. Prior studies have shown that the incidence is increasing in Western countries [2-4]. However, it has not been clearly identified at-risk subpopulations or regional trends [1]. The only curative treatment for cholangiocarcinoma is surgical resection; however, nearly two-thirds of cholangiocarcinoma are clinically silent and present at advanced stages with poor prognosis [2, 5]. Thus, there is a necessity to identify at-risk populations and regional variations for this cancer so that it can be diagnosed as early as possible, have the best chance for treatment, and ultimately improve survival. Prior studies have used the incidence rates from the National Cancer Institute’s (NCI) Surveillance, Epidemiology and End Results (SEER) program. The SEER database, however, only represents approximately 28% of the US population [6]. Consequently, the SEER database can under-represent certain racial/ethnic groups and regions in the USA [7]. The United States Cancer Statistics (USCS) combines both the Center of Disease Control and Prevention’s (CDC) National Program of Cancer Registries (NPCR) and the SEER database to include data for all 50 states and can have a much better representation of the US population [8]. In this study, we performed a comprehensive analysis of how the incidence of cholangiocarcinoma has changed over the years for different subpopulations in all 50 states.

## Materials and methods

Incidence data for cholangiocarcinoma, between 2001 and 2015, were obtained from the USCS database [9]. The USCS database provides official federal statistics on cancer incidence and population data for all 50 states and the District of Columbia [7]. International Classification of Diseases (ICD) for Oncology, third edition code data was extracted for cholangiocarcinoma (8160/3). Incidence data were stratified based on sex, race, stage, primary location (intrahepatic bile duct or extrahepatic bile duct), and US regions (Northeast, Midwest, South, and West). Stage was subclassified as localized, regional, and distant disease. Incidence analysis used Tiwari et al.’s (2006) modifications for confidence interval [10]. The Joinpoint Regression Program (version 4.5.0.1, DigitCompass LLC, MD, USA) was used to generate incidence curves and calculate annual percentage change (APC) using the least square [11]. Incidence data are per 100,000 and were adjusted to the year 2000 US standard population. For all analysis, p < 0.05 was considered statistically significant.

## Results

A total of 61,388 patients were included in the incidence analysis between 2001 and 2015 (Table 1). There were a total of 31,232 (50.9%) males and 30,156 (49.1%) females. There were a total of 60,612 patients with an identifiable race at the time of diagnosis. Of those, 51,429 (84.8%) were whites, 5,633 (9.3%) were blacks, and 3,550 (5.9%) were API. There were a total of 51,379 patients with an identifiable stage at diagnosis. Of those, 11,794 (22.9%) were localized, 18,944 (36.9%) were regional, and 20,641 (40.2%) were distant. There were a total of 4,957 patients with a primary site of either intrahepatic bile duct or extrahepatic bile duct. Of those, 3,315 (66.9%) were located in the intrahepatic bile duct while 1,642 (33.1%) were located in the extrahepatic bile duct. There were a total of 61,388 with an identified regional location within the USA at time of diagnosis. Of those, 13,340 (21.7%) were in the Northeast, 13,247 (21.6%), were in the Midwest, 20,850 (34%) were in the South, and 13,951 (22.7%) were in the West.

**Table 1:** Patient characteristics.

Patient characteristics		
Gender (n = 61,388)		
	Count	Percent
Male	31,232	50.9%
Female	30,156	49.1%
Race (n = 60,612)		
	Count	Percent
White	51,429	84.8%
Black	5,633	9.3%
Asian or Pacific Islander	3,550	5.9%
Stage (n = 51,379)		
	Count	Percent
Localized	11,794	22.9%
Regional	18,944	36.9%
Distant	20,641	40.2%
Primary site (n = 4,957)		
	Count	Percent
Intrahepatic bile duct	3,315	66.9%
Extrahepatic bile duct	1,642	33.1%
Regions (n = 61,388)		
	Count	Percent
Northeast	13,340	21.7%
Midwest	13,247	21.6%
South	20,850	34.0%
West	13,951	22.7%

**Figure 1:**
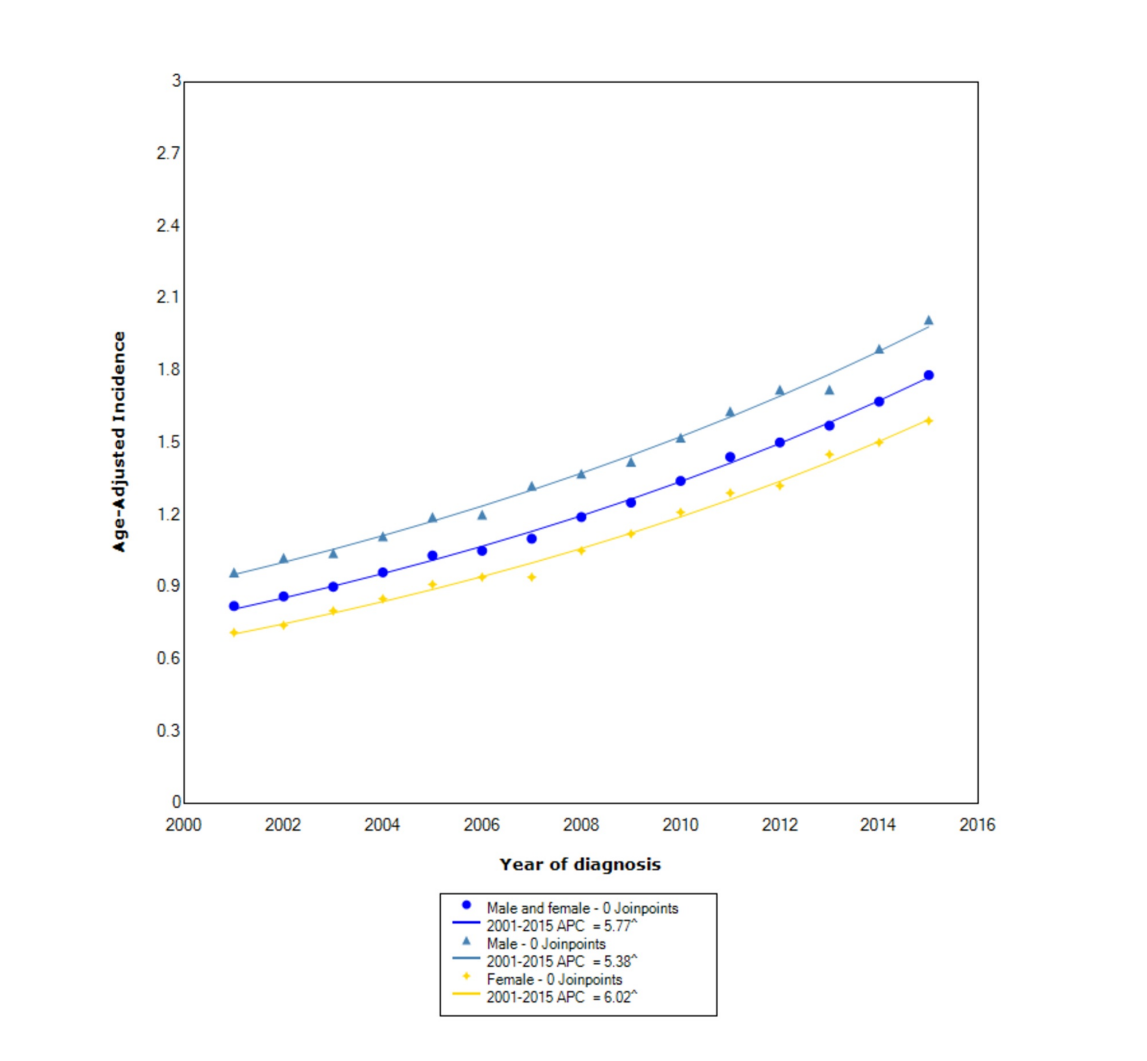
Incidence rate, sex. APC, annual percent change. ^ indicates that the APC is significantly different from zero at the alpha = 0.05. Age-adjusted incidences are per 100,000 and age adjusted to the 2000 US standard population.

The overall incidence of cholangiocarcinoma from 2001 to 2015 was 1.26 per 100,000 people per year. Males had an incidence of 1.44 (95% CI 1.42-1.46) which was slightly greater than the incidence in females which was 1.11 (95% CI 1.10-1.13). Between 2001 and 2015, the incidence in both males and females continued to increase at an exponential statistically significant rate with an APC of 5.38 and 6.02, respectively (Figure 1).

**Figure 2:**
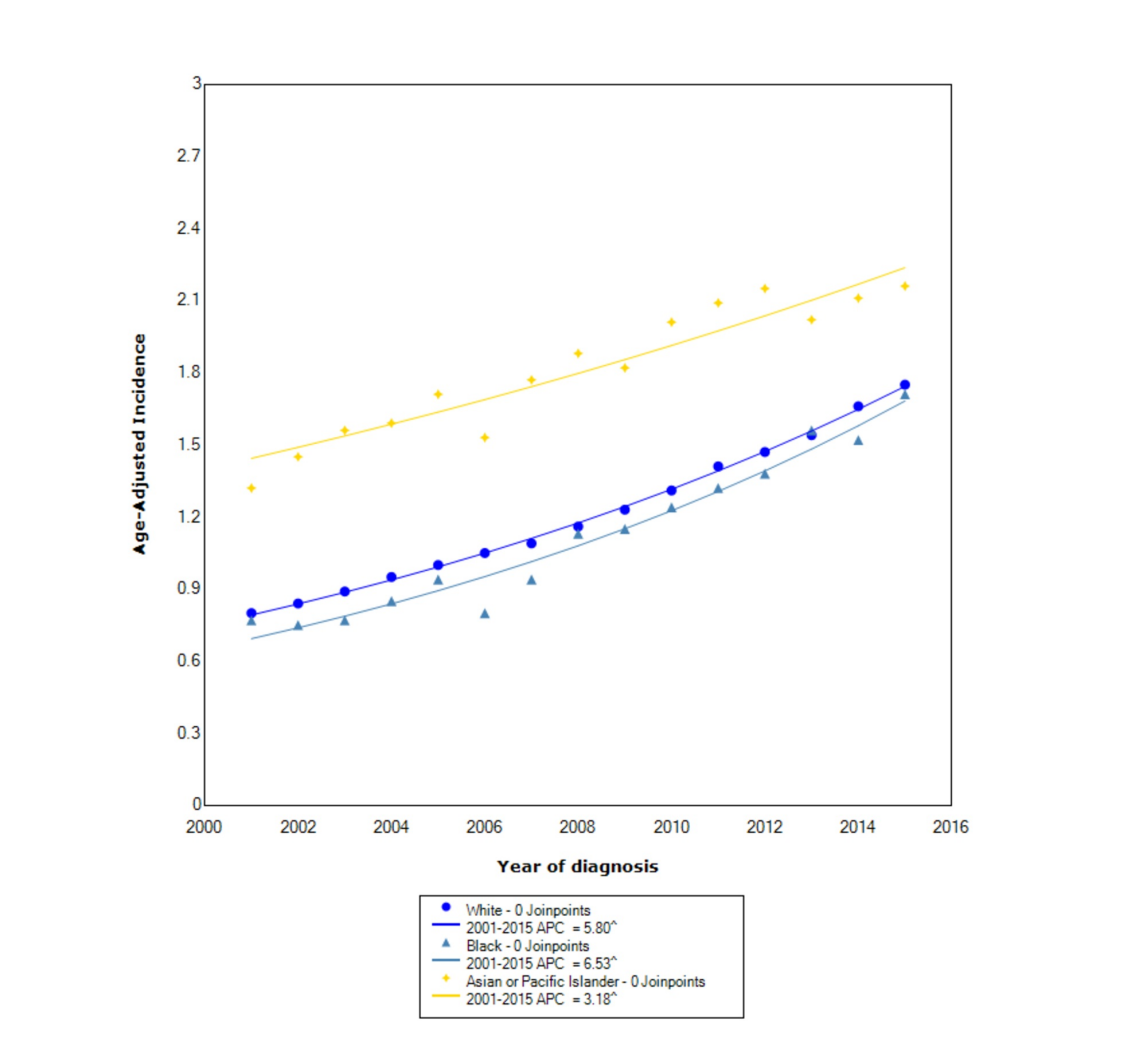
Incidence rate, race. APC, annual percent change. ^ indicates that the APC is significantly different from zero at the alpha = 0.05. Age-adjusted incidences are per 100,000 and age adjusted to the 2000 US standard population.

When stratified by race, cholangiocarcinoma had the greatest overall incidence in APIs (1.87 95% CI 1.81-1.94), followed by whites (1.23 95% CI 1.22-1.24), and lastly blacks (1.17 95% CI 1.13-1.20). Between 2001 and 2015, the incidence in APIs increased with statistical significance (APC 3.18). In whites, between 2001 and 2015, the incidence also increased at a statistically significant rate (APC 5.80). Of all the races, the incidence in blacks increased at the greatest statistically significant rate between 2001 and 2015 (APC 6.53) (Figure 2).

**Figure 3:**
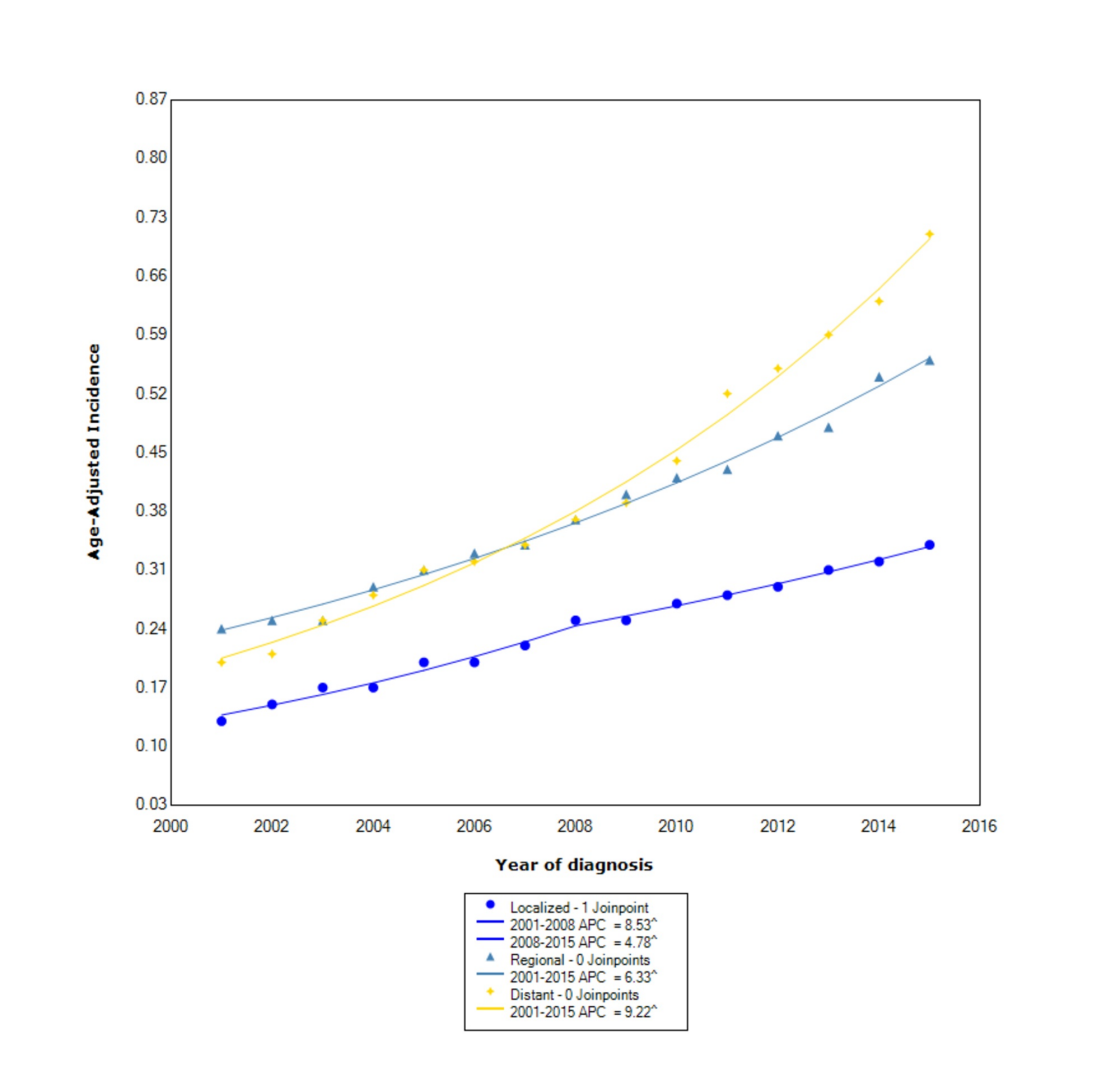
Incidence rate, stage. APC, annual percent change. ^ indicates that the APC is significantly different from zero at the alpha = 0.05. Age-adjusted incidences are per 100,000 and age adjusted to the 2000 US standard population.

When stratified by stage at diagnosis, cholangiocarcinoma had the greatest overall incidence in those with distant disease (0.42 95% CI 0.41-0.43), followed by regional disease (0.387 95% CI 0.381-0.393), and lastly localized disease (0.242 95% CI 0.238-0.247). During this time, the incidence increased at the greatest rate for those with distant disease (APC 9.22, statistically significant). Between 2001 and 2015, the incidence of regional disease increased with statistical significance (APC 6.33). For localized disease, between 2001 and 2008, the incidence increased with statistical significance (APC 8.53); however, after 2008 the incidence continued to rise with statistical significance, but at a slower rate (APC 4.78) (Figure 3).

**Figure 4:**
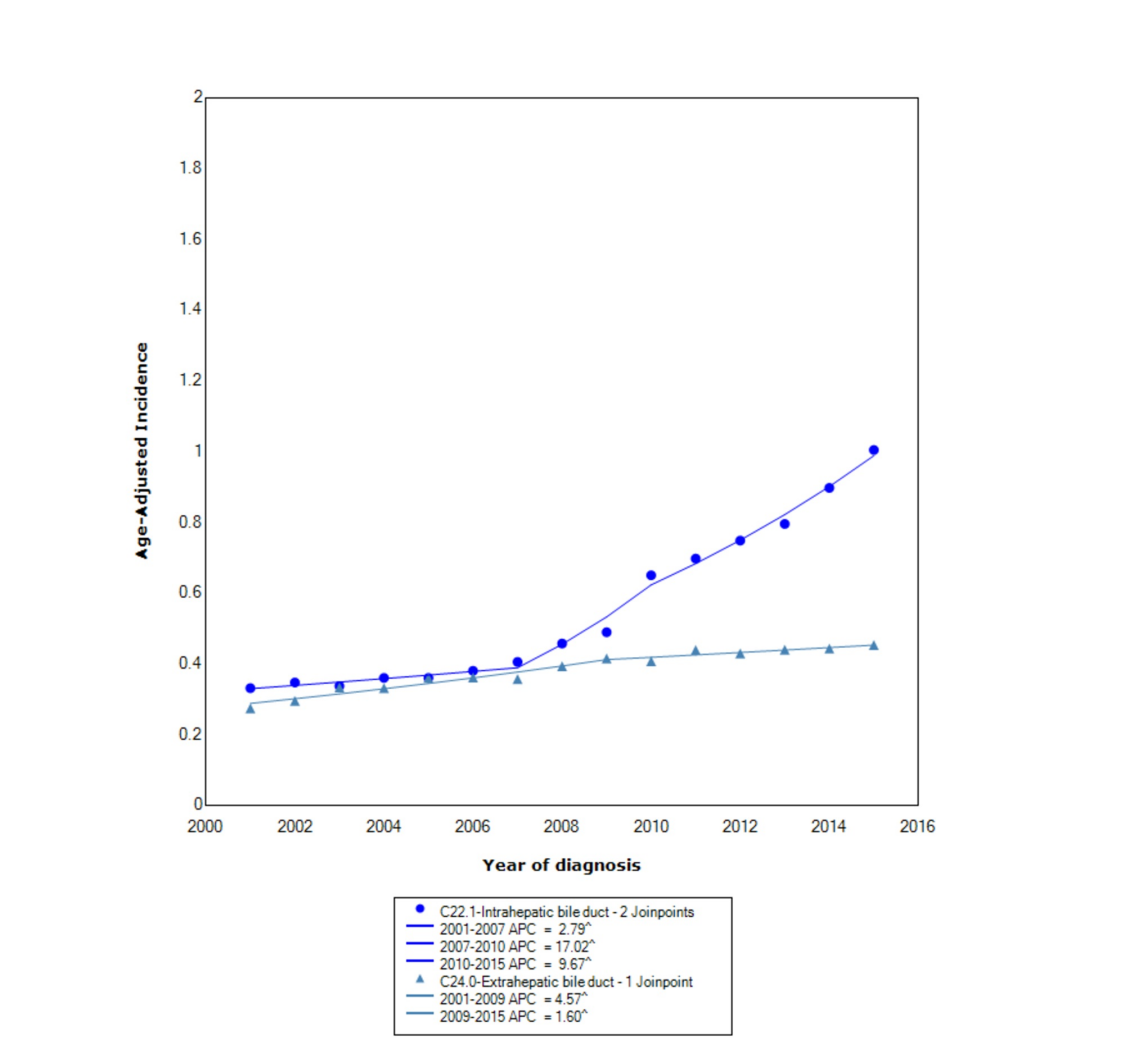
Incidence rate, primary site. APC, annual percent change. ^ indicates that the APC is significantly different from zero at the alpha = 0.05. Age-adjusted incidences are per 100,000 and age adjusted to the 2000 US standard population.

When stratified based on primary location, intrahepatic or extrahepatic bile duct, the overall incidence was greater in the intrahepatic bile duct (0.90 95% CI 0.87-0.92) than the extrahepatic bile duct (0.44 95% CI 0.42-0.46). Between 2001 and 2015, the incidence of ICC increased at an exponential rate compared to ECC. For ICC, between 2001 and 2007, the incidence increased with statistical significance (APC 2.79). Between 2007 and 2010, the incidence rose dramatically with a statistically significant APC of 17.02. After 2010, the incidence also increased with statistical significance but not at nearly the same rate (APC 9.67). For ECC, between 2001 and 2009, the incidence increased with statistical significance (APC 4.57); however, after 2009 the rise in incidence leveled off with a statistically significant APC of 1.60 (Figure 4).

**Figure 5:**
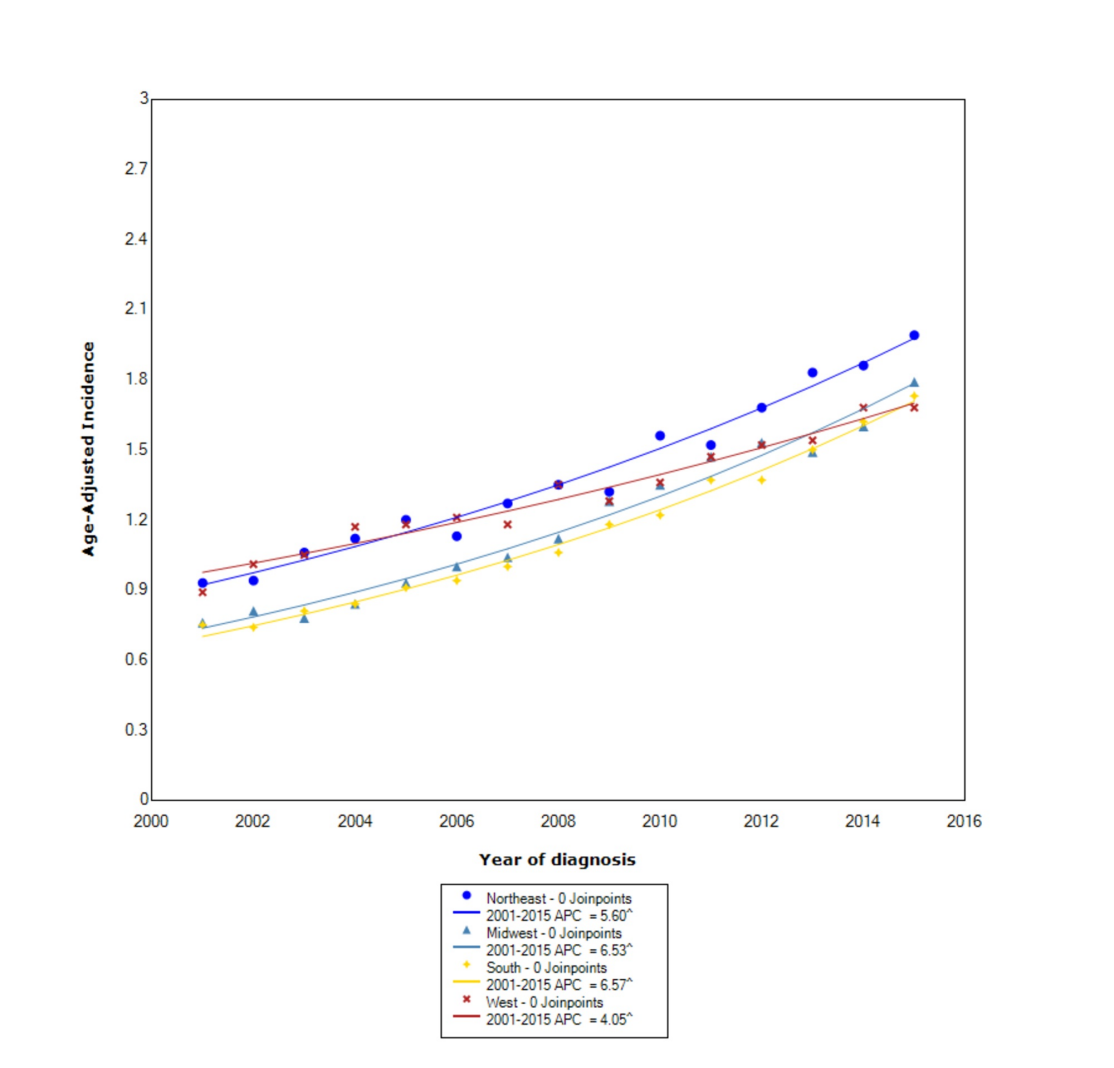
Incidence rate, region. APC, annual percent change. ^ indicates that the APC is significantly different from zero at the alpha = 0.05. Age-adjusted incidences are per 100,000 and age adjusted to the 2000 US standard population.

The incidence of cholangiocarcinoma stratified by regional location within the USA showed that the overall incidence was greatest in the Northeast (1.40 95% CI 1.37-1.42), followed by the West (1.33 95% CI 1.31-1.35), the Midwest (1.20 95% CI 1.18-1.22), and lastly the South (1.16 95% CI 1.15-1.18). Between 2001 and 2015, the incidence was increasing at the greatest rate with statistical significance for those in the South (APC 6.57). The other three regions, the Midwest, the Northeast, and the West were also all increasing with statistical significance between 2001 and 2015 with APCs of 6.53, 5.60, and 4.05, respectively (Figure 5).

## Discussion

Prior studies have shown an increased incidence of cholangiocarcinoma in Western countries; however, there is limited data on which subpopulations are at the greatest risk and if there are any regional variations [1-4]. Potential risk factors include parasitic infections, biliary duct cysts, primary sclerosing cholangitis (PSC), hepatolithiasis, inflammatory bowel disease, hepatitis C and B virus infections, cirrhosis, obesity, alcohol use, smoking, and diabetes [12]. In our study, we found a substantially increased incidence of ICC compared to ECC. We found an overall 2.05:1 ICC to ECC incidence ratio. Starting in 2007, the incidence of ICC started to increase at a significantly greater rate with an APC of 17.02 between 2007 and 2010 and an APC of 9.67 thereafter. Comparatively, the incidence of ECC increased with an APC of only 4.57 between 2001 and 2009 and an APC of 1.60 after 2009. The reason for these findings may be related to an improved diagnosis of ICC from cancers that were once diagnosed as cancers of unknown primary (CUP) [1]. In the past, ICC would be misdiagnosed as metastatic disease from another primary or CUP due to the fact that ICC share many of the same immunohistochemistry (IHC) with other cancers, such as staining positive for cytokeratin (CK) seven and CK19 and negative staining for CK20 [1, 13]. Moreover, the tumor markers for ICC are nonspecific and include carcinoembryonic antigen (CEA) and carbohydrate antigen (CA) 19-9 [1, 14].

In our study, we also found that APIs had the greatest overall incidence rate when compared to other races. Prior studies have also demonstrated that Asians are more likely to be diagnosed with cholangiocarcinoma [12]. However, we also found that the incidence is increasing at a significant rate in all races in the USA and not just APIs. Given the worsening incidence in all races, it is difficult to identify one specific risk factor leading to our findings. Likely, there are multiple risk factors affecting different subpopulations at different percentages. For example, hepatitis B infection has been shown to be a risk factor for ICC in Asia whereas in Western countries, like the USA, hepatitis C has been shown to be a risk factor [2]. Moreover, in the USA, along with PSC, which is likely the most common risk factor for cholangiocarcinoma, rates of obesity, diabetes, alcohol use are all rising and likely are all significantly impacting the incidence of cholangiocarcinoma [1, 12].

Similar to our findings of a rising incidence in all races, we also found that the incidence in all four regional locations of the USA are increasing, without one region increasing at a more rapid rate than another. This finding demonstrates that the risk factors for cholangiocarcinoma are not regional and are likely impacted by the same factors we described above for races. Ultimately, the impact hepatitis B or C viral infection, diabetes, obesity, PSC, alcohol intake, or even other potential risk factors such as diet, environmental carcinogen exposure, and medication have on the incidence of cholangiocarcinoma is poorly understood and require further risk factor specific investigation. Our study, however, demonstrates that these risk factors are not just affecting one specific subpopulation but are having an effect on all of them.

When evaluating the incidence of cholangiocarcinoma by stage at the time of diagnosis, the overall incidence was greatest for distant disease. Moreover, between 2001 and 2015, the rise in incidence was also the greatest in those with distant disease, with an APC of 9.22. Cholangiocarcinoma is a silent disease and presents typically at advanced stages. Our findings are alarming and only validate our concern about the rising incidence of cholangiocarcinoma and the need for further research on risk factors affecting the disease so that we can diagnose this cancer as early as possible and ultimately improve survival and reduce mortality.

There are several strengths and limitations to our study. One strength of this study was the use of the USCS database which includes data for all 50 states in the USA [7]. Prior studies have used the SEER database which is limited by having data on only a limited percentage of the US population and thus likely does not represent the true incidence of cancers as well as the USCS database [7]. Another strength of our study was the identification of at-risk subpopulation. A limitation of our study was the inability to identify specific causality or correlations between specific risk factors and those being affected by the disease. Our findings showed that the incidence of cholangiocarcinoma is increasing in nearly every subpopulation at an alarming rate, and thus the need for further research is highly warranted.

## Conclusions

In our study, we evaluated the incidence of cholangiocarcinoma in all 50 states. For each of the stratifications we evaluated, the overall incidence was greatest in males, APIs, distant disease, ICC, and those in the Northeast; however, the incidence was increasing in all subpopulations. The reasons for these trends are unclear and require further studies. Our study, to the best of our knowledge, is the first to evaluate the incidence of cholangiocarcinoma, stratified by different subpopulations, in all 50 states. Ultimately, our findings will help identify patients that are at high risk for cholangiocarcinoma but further studies are needed to help reduce the burden of this fatal cancer.
